# P-1600. Relationships between Anti-spike SARS-CoV-2 IgG Antibodies at Delivery, Timing of COVID-19 Vaccine Booster Doses in Pregnancy and Risk of Maternal and Infant Infections – The UK Preg-CoV Study

**DOI:** 10.1093/ofid/ofaf695.1779

**Published:** 2026-01-11

**Authors:** Eva P Galiza, Natalie Marchevsky, Xinxue Liu, Paul T Heath

**Affiliations:** City St. George's, University of London, London, England, United Kingdom; Centre for Clinical Vaccinology and Tropical Medicine (CCVTM), University of Oxford, Oxford, England, United Kingdom; Centre for Clinical Vaccinology and Tropical Medicine (CCVTM), University of Oxford, Oxford, England, United Kingdom; Vaccine Institute, Centre for Neonatal and Paediatric Infection, St George’s University of London, London, England, United Kingdom

## Abstract

**Background:**

The COVID-19 pandemic had a significant impact on pregnant women and infants, yet few prospective COVID-19 vaccine studies were undertaken. The Preg-CoV study examined the safety, immunogenicity and optimal timing of COVID-19 vaccine doses in a UK pregnancy cohort.

**Table 1.**
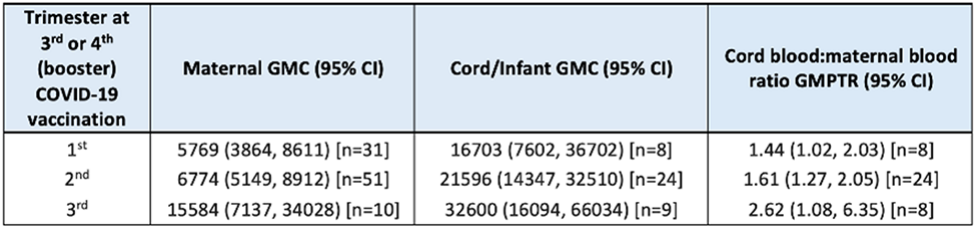
Maternal and cord/infant anti-spike SARS-CoV-2 IgG geometric mean concentrations (GMC), and placental transfer ratios (cord/infant blood:maternal blood ratio) by trimester of COVID-19 booster dose received during pregnancy.

**Figure 1.**
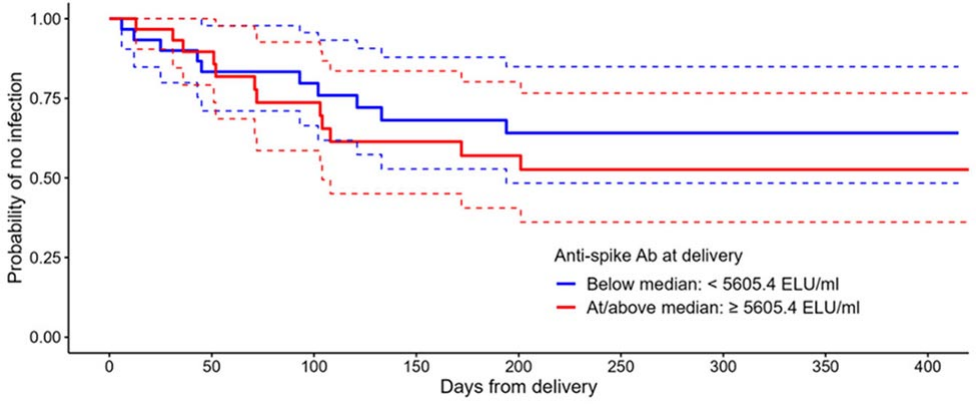
Kaplan-Meier curve for maternal infection post-delivery by anti-spike SARS-CoV-2 IgG antibody at delivery, below and above the median level (5605.4 ELU/ml). Includes participants receiving one COVID-19 dose during pregnancy as 3rd or 4th dose (booster). Excludes participants with anti-nucleocapsid evidence of infection without self-reported swab, participants with positive swab prior to delivery, participants with no anti-spike SARS-CoV-2 IgG at delivery. Participants are censored at the earliest date of withdrawal, loss to follow-up, end of study, or vaccination after delivery.

**Methods:**

The Preg-CoV study is a Phase II, multi-centre, hybrid randomised and observational platform study of pregnant women and their infants. Participants from 13 sites were recruited Aug 2021 - Oct 2022. Maternal and cord/infant blood samples were taken at delivery or within 3 days of birth and anti-spike SARS-CoV-2 IgG geometric mean concentrations (anti-S IgG GMC, ELISA) and geometric mean placental transfer ratios (GMPTR) were measured. Episodes of SARS-CoV-2 infection (nasopharyngeal swabs, PCR/lateral flow) up to 12 months post-delivery were recorded.

**Figure 2.**
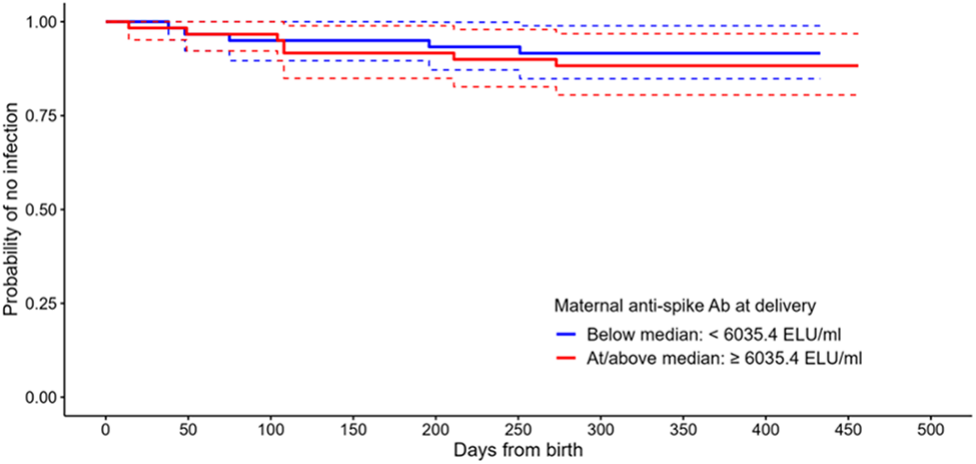
Kaplan-Meier curve for infant infection by maternal anti-spike SARS-CoV-2 IgG antibody at delivery, below and above the median level (6035.4 ELU/ml). Includes infants whose mothers received one COVID-19 dose during pregnancy as 3rd or 4th dose (booster). Excludes infants with no maternal anti-spike SARS-CoV-2 IgG at delivery. Infants censored at the earliest date of withdrawal, loss to follow-up, end of study.

**Results:**

317 (309 evaluable) participants were recruited. Median age at enrolment was 34 years (interquartile range [IQR] 30.4-36.7), median gestational age (GA) 28^+4^ weeks [23^+0^- 34^+3^], 54% had co-morbidities and 38% were SARS-CoV-2 seropositive; median GA at birth was 39^+3^ weeks [38^+6^- 40^+4^] and 36% delivered by caesarean section. 183 women received a 3^rd^ or 4^th^ COVID-19 vaccine booster dose during pregnancy whose GMPTR and cord/infant blood anti-S IgG levels at delivery were similar between trimester at receipt of their COVID-19 vaccine booster dose with an increased trend when the booster was given later in pregnancy (table 1). Of these participants, 32 (17%) had a positive swab antenatally and 26 (14%) postnatally. Of the 175 infants born to these women, 13 (7%) recorded a positive swab up to 12 months of age. Kaplan-Meier curves for postnatal infections (fig 1&2) demonstrate no significant association between maternal anti-S IgG antibody levels at delivery and COVID-19 infections. No COVID-19-related hospitalisations or deaths occurred.

**Conclusion:**

Placental transfer of anti-S IgG antibodies is high ( > 1) across all trimesters, supporting a flexible maternal vaccination schedule, although trends suggest increased responses when a booster is given later in pregnancy. There was no evidence of a relationship between maternal anti-S IgG antibody level at delivery and subsequent mild infection rates in mothers or infants.

**Disclosures:**

All Authors: No reported disclosures

